# Comparative transcriptomic analysis revealed dynamic changes of distinct classes of genes during development of the Manila clam (*Ruditapes philippinarum*)

**DOI:** 10.1186/s12864-022-08813-0

**Published:** 2022-09-29

**Authors:** Yanming Zhang, Hongtao Nie, Zhihui Yin, Xiwu Yan

**Affiliations:** 1 College of Fisheries and Life Science, Dalian Ocean University, 116023 Dalian, China; 2 Engineering Research Center of Shellfish Culture and Breeding in Liaoning Province, College of Fisheries and Life Science, Dalian Ocean University, 116023 Dalian, China

**Keywords:** Different developmental stages, Molecular basis, *Ruditapesphilippinarum*, Transcriptomics

## Abstract

**Background:**

The Manila clam *Ruditapesphilippinarum* is one of the most economically important marine shellfish. However, the molecular mechanisms of early development in Manila clams are largely unknown. In this study, we collected samples from 13 stages of early development in Manila clam and compared the mRNA expression pattern between samples by RNA-seq techniques.

**Results:**

We applied RNA-seq technology to 13 embryonic and larval stages of the Manila clam to identify critical genes and pathways involved in their development and biological characteristics. Important genes associated with different morphologies during the early fertilized egg, cell division, cell differentiation, hatching, and metamorphosis stages were identified. We detected the highest number of differentially expressed genes in the comparison of the pediveliger and single pipe juvenile stages, which is a time when biological characteristics greatly change during metamorphosis. Gene Ontology (GO) enrichment analysis showed that expression levels of microtubule protein-related molecules and Rho genes were upregulated and that GO terms such as ribosome, translation, and organelle were enriched in the early development stages of the Manila clam. Kyoto Encyclopedia of Genes and Genomes pathway analysis showed that the foxo, wnt, and transforming growth factor-beta pathways were significantly enriched during early development. These results provide insights into the molecular mechanisms at work during different periods of early development of Manila clams.

**Conclusion:**

These transcriptomic data provide clues to the molecular mechanisms underlying the development of Manila clam larvae. These results will help to improve Manila clam reproduction and development.

**Supplementary Information:**

The online version contains supplementary material available at 10.1186/s12864-022-08813-0.

## Background

Mollusks, which can be aquatic or terrestrial, are the most morphologically diverse group of invertebrate species. However, this phylum exhibits the conserved and characteristic developmental processes of spiral cleavage and trochophore larvae. The early development of mollusks consists of a series of developmental periods during which changes in body form and lifestyle occur [1]. Bivalves are the second largest group in the phylum of Mollusca with regard to species number [2]. Bivalves include almost all economically important shellfish (i.e., oysters, scallops, clams, and mussels). The morphology of the bivalves changesdramatically during early development, and their lifestyle changes from planktonic to benthic or sessile [3–5]. Therefore, studies of the molecular processes during early development of bivalves will provide the basis for understanding the evolution and early diversification of animals [6].

The body morphology and lifestyle of bivalves change during development from larvae to adults, and many genes are involved in the transformation from free-swimming to benthic larvae and in the metamorphosis stage [7]. The expression of caveolin, tubulin and tektin provides a reference for the early development of ciliary bands, which are the first organs to emerge during molluscan embryonic differentiation [8]. Tyrosinase may be involved in the biosynthesis of non-calcified shells in Pacific oyster *Crassostrea gigas* [9]. Dopamine receptor genes may be involved in the larvae shell formation in Pacific oyster *C. gigas* [10]. In the *Meretrix meretrix*, mRNA differential expression analysis showed that expression of the MmeFer gene in larvae increased at least 8-fold after the trochophore stage, indicating that it played an important role in the calcification process of the shell in this species [11].

Larval metamorphosis in most bivalves is accompanied by high mortality due to both external and internal factors [12]. A few receptor genes, functional genes, and chemical cues control the metamorphosis of planktonic larvae to benthic larvae [13]. For example, steroid receptors identified by combined multiomics analysis may play an important role during larval metamorphosis in pearl oyster(*Pinctada fucata martensii*) [14]. In the Pacific oyster, myosin heavy chain knockdown was found to regulate proper myoblast assembly during larval development [15]. Caspase gene expression and in situ hybridization results suggested an important role of this gene in the process of veliger shedding during larval metamorphosis of the Fujian oyster (*Crassostreaangulata*) [16], and it may also be involved in larval metamorphosis of *Meretrix* via the apoptotic pathway [17]. The specific expression pattern of molluscan growth and differentiation factor in *C. gigas* suggested that this factor may play a central role in the metamorphosis process of oyster larvae [18]. The N-methyl-D-aspartate pathway has a recognized regulatory function in the metamorphosis of the Pacific oyster [19], and many neuroactive compounds, including γ-aminobutyric acid, various catecholamines, 5-hydroxytryptamine, and nitric oxide (NO) are associated with metamorphosis in various mollusks [20].

The Manila clam is widely distributed in the sandy mud sediments of tidal flats and shoals along the coasts of China, the northwestern United States, and the Atlantic coast of Europe. It is an economically important marine bivalve, with a production of 4.2 million tons in 2020 [21]. Due to its economic importance and ecological role, many researchers have studied the genetics, breeding, and disease control of this species. Fertilization and hatching rates are important indicators in artificial breeding programs because it has a significant impact on shellfish larval production [22]. Studies have shown that the production of clam juvenile is inextricably linked to hatching rate and larval mortality [23], and it has been demonstrated that functional genes play important roles on the hatching and development process [24]. Understanding the regulatory steps at work during the different stages of early development is critical to artificial breeding and developmental biology research. The growth traits variability and developmental process of the Manila clam has been described in our previous study [25, 26], however, the dynamic changes of distinct classes of genes during embryo and larvae development of the Manila clam is still largely unexplored. In this study, we focused on the molecular processes involved in different developmental stages of Manila clam. Our comprehensive analysis of gene regulation during early development provided a framework for understanding the developmental dynamics of the Manila clam.

The goals of this study were to reveal the molecular mechanisms involved in the early development of the Manila clam and to elucidate the relationship between gene expression and morphological alterations. To investigate the transcriptional processes that occur during clam development, we used RNA-seq to explore the genetic basis of and regulatory genes involved in early development of the Manila clam and to uncover important biological processes, molecular functions and developmental regulation of gene expression during ontogeny.

## Materials and methods

Manila clam was collected from the Dalian, Liaoning Province, China, and the gonadal maturity of the population was assessed by regular sampling. After the clams spawned, we mixed sperm with eggs while stirring clockwise to allow full fertilization until fertilized eggs (FE) could be observed (Fig. S1). Samples were also collected and stored for each of the remaining stages. After 0–15 min, the number of first polar body was > 90% (PB1), and specimens were collected and stored in liquid nitrogen. After 20–44 min, the number of second polar body was > 90% (PB2), and specimens were sampled and stored in liquid nitrogen. After 1 h, the eggs entered the two-cell stage (TC), after 84–107 min it entered the eight-cell stage (EC), after 160–180 min it entered the blastula stage (B), and from 3 to 6 h it entered the gastrula stage (G),and then hatched to become trochophore larvae (T), followed by D-pattern larvae (D) after 1 day and umbo-veliger larvae (U) after 2–5 days. After 15 days, the larvae entered the pediveliger larval stage (P). The larvae metamorphosed between 19 and 21 days and grew from a single pipe juvenile (S) to a double plumbed juvenile (J) (Table S1). All methods were carried out in accordance with relevant guidelines and regulations.

### Transcriptome sequencing and assembly

Total RNA samples were extracted, and their quality was tested using an Agilent 2100 bioanalyzer. The mRNA was then enriched, and the library was built using the NEBNext® Ultra™ Directional RNA Library Prep Kit for Illumina®, operating on 42 sets of samples. After library construction, the libraries (diluted to 1.5 ng/µl) were initially quantified using a Qubit 2.0 Fluorometer and subsequently tested for insert size using the Agilent 2100 bioanalyzer. For insert sizes that met expectations, qRT-PCR was performed to quantify the effective concentration of the library. The libraries were then accurately quantified by qRT-PCR (effective library concentration above 2 nM) to ensure library quality.

### Quality control and reads mapping to the reference genome

Analysis of raw image data files generated by the Illumina HiSeq 2500 instrument, where sequenced fragments were converted into sequence data (reads) by the high-throughput sequencer with CASAVA base identification. The raw reads were processed using an internal Perl script [27]. In this step reads with adapters were removed; reads containing N were removed (N indicates that base information could not be determined), and low-quality reads (reads with Qphred < = 20 bases accounted for more than 50% of the entire read length) were removed. After raw data filtering, sequencing error rate checking and GC content distribution checking, clean reads were obtained for subsequent analysis. At the same time, Q20, Q30 and GC content were calculated for the clean data. All downstream analyses were performed using high quality (> Q30) reads. Reference genome and gene model annotation files were obtained from Yan et al. (2019) [28]. Reference genomes were indexed using Hisat2 v2.0.5 and paired clean reads were aligned to the reference genome using Hisat2 v2.0.5 [29]. The new transcripts were assembled by String Tie software [30], using a network flow algorithm together with optional de novo assembly, to assemble these complex data sets into transcripts.

### Quantification of gene expression levels

**Fig. 1:**
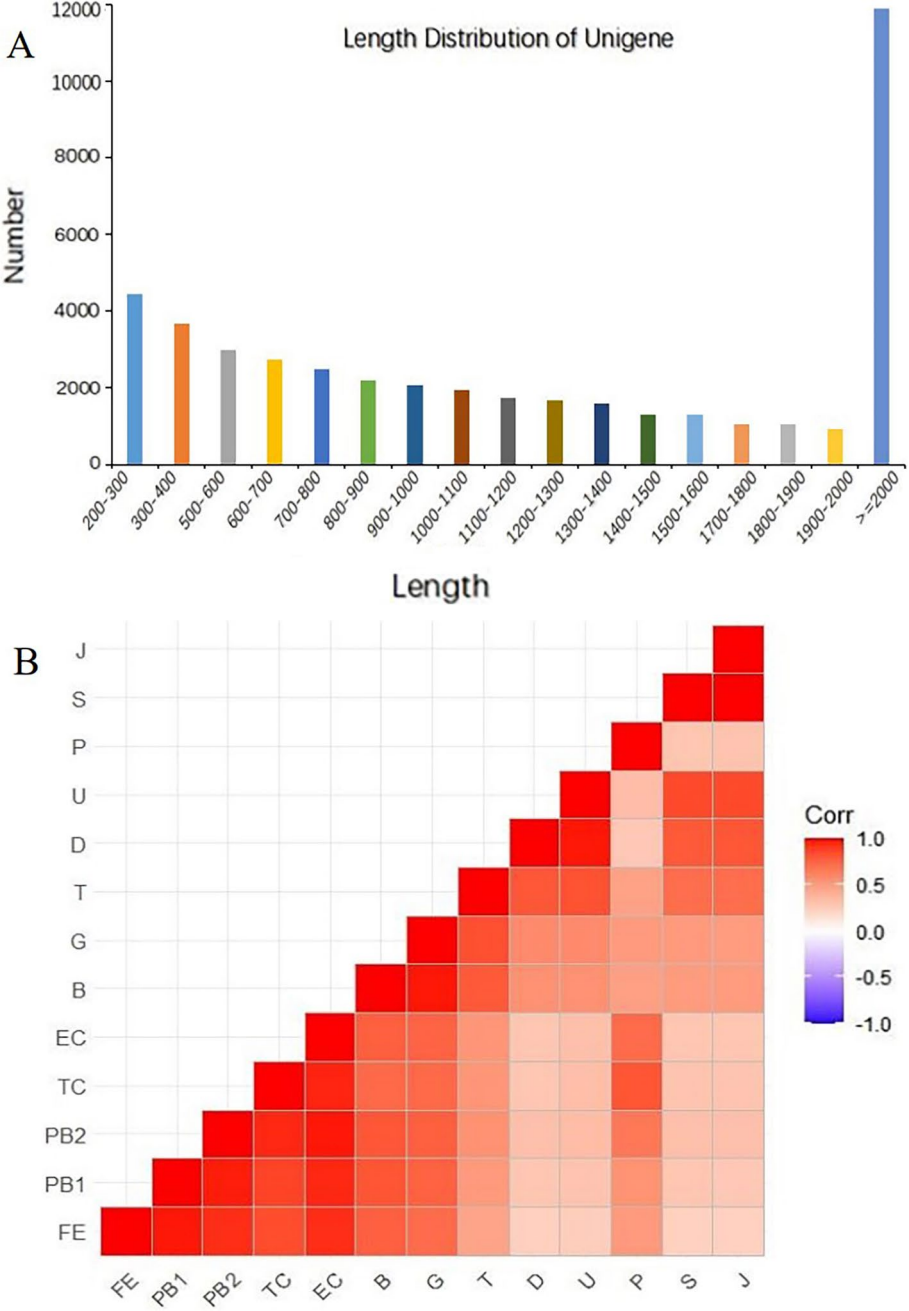
Assembly and information about the transcriptome data of the Manila clam. **A** Length distribution of unigenes after assembly; the abscissa represents the length range of unigenes, and the ordinate represents the number of unigenes corresponding to the length. **B** Visualization of Pearson correlation analysis of samples from the 13 developmental stages

The aims of this process were to count the number of reads for the full range of all genes using the Feature Counts tool in the subread 1.6.4 software [31], to match positions on the reference genome to genes, to filter out reads with low quality, to filter out reads from overlapping regions, and to match non-matched reads to multiple regions. The fragments per kilobase of transcript per million mapped reads (FPKM) is a common metric for estimating gene expression levels, and it is corrected for sequencing depth and sequencing length. Thus, the use of FPKM reduces the effect of gene length and sequencing depth on reads count, and it is generally considered to have a threshold value of 1. The resulting FPKM values were analyzed by principal component analysis (PCA). The expression of differential genes was clustered using the H-cluster method. To visualize the correlation of the samples, we created a correlation coefficient plot (Fig. 1B).

### Differential gene expression analysis

Differential expression analysis of samples within groups was performed using DESeq2 software [32]. It uses a model based on a negative binomial distribution to provide statistical routines for differential expression of gene expression data. The *p*-values obtained are used to control for false discovery rates to reduce the proportion of false positives using the Benjamini-Hochberg method for p-value correction. *P*-value (padj) < 0. 05, |log2foldchange| > 0 is generally considered to be the criterion for differential genes.

### Analysis of gene enrichment

We performed KEGG pathway enrichment analysis on the differential gene sets using clusterProfile (R 3.4.3) software [33], which is a comprehensive database that integrates genomic, chemical, and phylogenetic information. KEGG pathway enrichment is based on padj < 0. 05 as the threshold for significant enrichment. Gene Ontology (GO) functionally significant enrichment analysis and genomic background comparison of differential expression of genes in GO functionally significant enrichment terms reveals differential expression of genes significantly associated with biological function (http://www.geneontology.org/). The WGCNA (weighted gene co-expression network analysis) algorithm is a typical systems biology algorithm for constructing gene co-expression networks based on high-throughput gene messenger RNA (mRNA) expression data and is widely used in the international biomedical field. GO and KEGG, WGCNA enrichment. The analysis of DEGs is implemented in the cluster Profiler R package (3.4.4), where a correction for gene length bias was applied. GO terms and KEGG pathways were considered significantly enriched for DEGs if padj < 0.05 of DEGs.

### Real-time quantitative PCR (RT-qPCR) for DEGs validation

To validate the RNA-Seq data, inhibitor of apoptosis protein (IAP) - Birc7-a, Caspase-7, Caspase-3, EGFR (Epidermal growth factor receptor), Grb2 (Growth factor receptor-bound protein 2) and MAPK signaling pathway gene - Map2k1, a total of six expressed genes (FPKM > 1) were selected for qRT-PCR. Among them, Caspase-7, Caspase-3 and Birc7-a were shown to play important roles in apoptosis [34, 35]. MAPK pathway was reported to have important roles in cell growth and differentiation [36], and EGFR signaling pathway plays important roles in physiological processes such as cell growth, proliferation and differentiation [37], and was detected in this study. Grb2, a growth-related candidate gene, was reported in *Charybdis japonica* [38]. Total RNA from FE, PB1, PB2, TC, EC, B, G, T, D, U, P, S, and J, samples of 30 mg was isolated using TRIzol with three biological replicates in each group. Primers were designed using Primer5 software [39] (Premier Biosoft International, Palo Alto, CA, USA) (Table S2). β-actin genes were used as an internal control for qRT-PCR analysis. Single-stranded cDNA was synthesized with a reverse transcription system using the reverse transcriptase kit “PrimeScript™ RT Kit” (TaKaRa Bio, Shiga, Japan) according to the manufacturer’s instructions.

qRT-PCR was performed using TB Green Premix ExTaqII (TaKaRa Biologicals, Shiga, Japan). The qRT-PCR was conducted in a final volume of 20 µL, which consisted of 1 µL of cDNA (50 ng/µL), 1 µL of forwarding primer (80 ng/µL), 1 µL of reverse primer (80 ng/µL), 7 µL of ddH_2_O, and 10 µL of TB Green, with polymerase activation at 94 ℃ for 5 min, followed by 40 cycles at 94 ℃ for 30 s, 60 ℃ for 30 s and 72 ℃ for 30 s. Each sample was processed in triplicate in the Roche LightCycler 480 Real Time PCR System (Roche, Basel, Switzerland). All data were analyzed using the 2^−ΔΔCt^ method.

## Results

### Transcriptome sequencing

We subjected samples from 13 different developmental stages (FE, PB1, PB2, TC, EC, B, G, T, D, U, P, S, and J) of Manila clams to RNA-seq (Table S3). A total of 1,127,214,960 raw reads and 1,082,461,572 clean reads (96.03% of raw reads) were acquired from the 39 RNA-seq libraries. After raw data filtering, sequencing error rate checking, and GC content distribution analysis, clean reads for subsequent analysis were obtained. This work yielded 16.1 Gb of data with Q20 > 97.11% and Q30 > 91. 65% (Table 1), for a total of 50,285 genes for differentially expressed gene (DEG) analysis. The distribution of sequence lengths indicated that most single genes were more than 2000 base pairs in length; the second highest number of single genes were between 200 and 300 base pairs in length (Fig. 1A). All genes had between 30% and 40% GC content (Fig. S2). The sample correlation matrix based on Pearson’s correlation coefficients (Fig. 1B) showed satisfactory correlation between samples from different developmental periods and between samples from adjacent periods. The transcriptome correlations of the sample collections were analyzed by hierarchical clustering and principal component analysis (PCA). PCA results revealed that the samples (FE, PB1, PB2, EC, TC, P), (S, J), (U, D), and (B, G) differed significantly from each other (Fig. S3).

**Table 1:** Summary of RNA-seq analysis based on the R. philippinarum transcriptomes

sample	library	raw_reads	clean_reads	clean_bases	error_rate	Q20	Q30	GC_pct
B1	FRAS202210859-1r	43,955,668	41,240,258	6.19G	0.03	97.01	91.4	36.97
B2	FRAS202210860-1r	45,773,354	43,133,392	6.47G	0.03	97.4	92.37	33.32
B3	FRAS202210861-1r	46,226,514	44,554,898	6.68G	0.03	97.05	91.57	33.06
D1	FRAS202210868-1r	45,021,880	43,356,444	6.5G	0.03	97.27	92.04	33.44
D2	FRAS202210869-1r	43,959,224	42,842,964	6.43G	0.03	97.01	91.38	33.87
D3	FRAS202210870-2r	44,267,138	42,584,080	6.39G	0.03	97.75	93.23	34.65
EC1	FRAS202210853-1r	46,129,118	43,964,100	6.59G	0.03	97.15	91.66	31.74
EC2	FRAS202210854-1r	40,342,868	39,191,980	5.88G	0.03	96.85	90.94	31.73
EC3	FRAS202210855-1r	46,780,072	44,927,680	6.74G	0.03	97	91.36	31.71
FE1	FRAS202210841-1r	47,326,730	45,422,278	6.81G	0.03	96.93	91.34	34.32
FE2	FRAS202210842-1r	44,260,130	42,490,688	6.37G	0.03	96.89	91.26	33.5
FE3	FRAS202210843-1r	46,776,748	44,882,736	6.73G	0.03	96.94	91.26	32.49
G1	FRAS202210862-1r	43,584,396	41,886,498	6.28G	0.03	97.22	91.91	32.83
G2	FRAS202210863-1r	43,219,570	41,582,452	6.24G	0.03	97.22	91.98	34.1
G3	FRAS202210864-1r	44,044,804	42,638,312	6.4G	0.03	97.15	91.74	32.73
J1	FRAS202210880-1r	47,022,484	45,390,096	6.81G	0.03	97.18	91.88	35.74
J2	FRAS202210881-1r	45,234,870	43,510,270	6.53G	0.03	97.17	91.78	34.81
J3	FRAS202210882-1r	45,850,112	43,860,432	6.58G	0.03	97.3	92.1	34.35
P1	FRAS202210874-1r	47,613,292	46,100,988	6.92G	0.03	97.05	91.36	33.7
P2	FRAS202210875-1r	45,571,546	44,192,698	6.63G	0.03	96.85	91.01	33.08
P3	FRAS202210876-1r	45,057,494	43,713,638	6.56G	0.03	97.11	91.57	33.66
PB1_1	FRAS202210844-1r	45,417,676	43,792,044	6.57G	0.03	96.72	90.82	32.83
PB1_2	FRAS202210845-1r	45,163,062	42,668,094	6.4G	0.03	96.96	91.29	32.97
PB1_3	FRAS202210846-1r	45,343,044	43,198,356	6.48G	0.03	97.11	91.69	33.14
PB2_1	FRAS202210847-1r	43,273,166	41,336,196	6.2G	0.03	97.17	91.78	32.22
PB2_2	FRAS202210848-1r	42,948,898	41,104,980	6.17G	0.03	96.97	91.34	32.26
PB2_3	FRAS202210849-1r	41,586,510	39,737,474	5.96G	0.03	97.11	91.61	32.15
S1	FRAS202210877-1r	44,572,994	43,317,056	6.5G	0.03	97.01	91.45	35.81
S2	FRAS202210878-1r	44,611,258	43,410,122	6.51G	0.03	97.18	91.86	35.14
S3	FRAS202210879-1r	44,270,558	43,022,476	6.45G	0.03	97.3	92.13	35.4
T1	FRAS202210865-1r	46,155,010	44,704,922	6.71G	0.03	97.03	91.45	32.19
T2	FRAS202210866-1r	46,228,808	44,959,292	6.74G	0.03	96.88	91.06	32.58
T3	FRAS202210867-1r	40,641,392	39,456,304	5.92G	0.03	96.94	91.2	32.31
TC1	FRAS202210850-1r	41,836,038	40,196,782	6.03G	0.03	96.98	91.22	31.96
TC2	FRAS202210851-1r	42,698,236	40,866,586	6.13G	0.03	97.18	91.78	32.1
TC3	FRAS202210852-1r	41,254,704	39,525,346	5.93G	0.03	97.45	92.23	31.83
U1	FRAS202210871-1r	47,866,654	46,187,404	6.93G	0.02	98.26	94.39	32.23
U2	FRAS202210872-1r	44,217,642	42,542,540	6.38G	0.03	96.86	91.08	33.81
U3	FRAS202210873-1r	42,624,270	41,253,684	6.19G	0.03	96.75	90.76	33.21

### DEG analysis

**Fig. 2:**
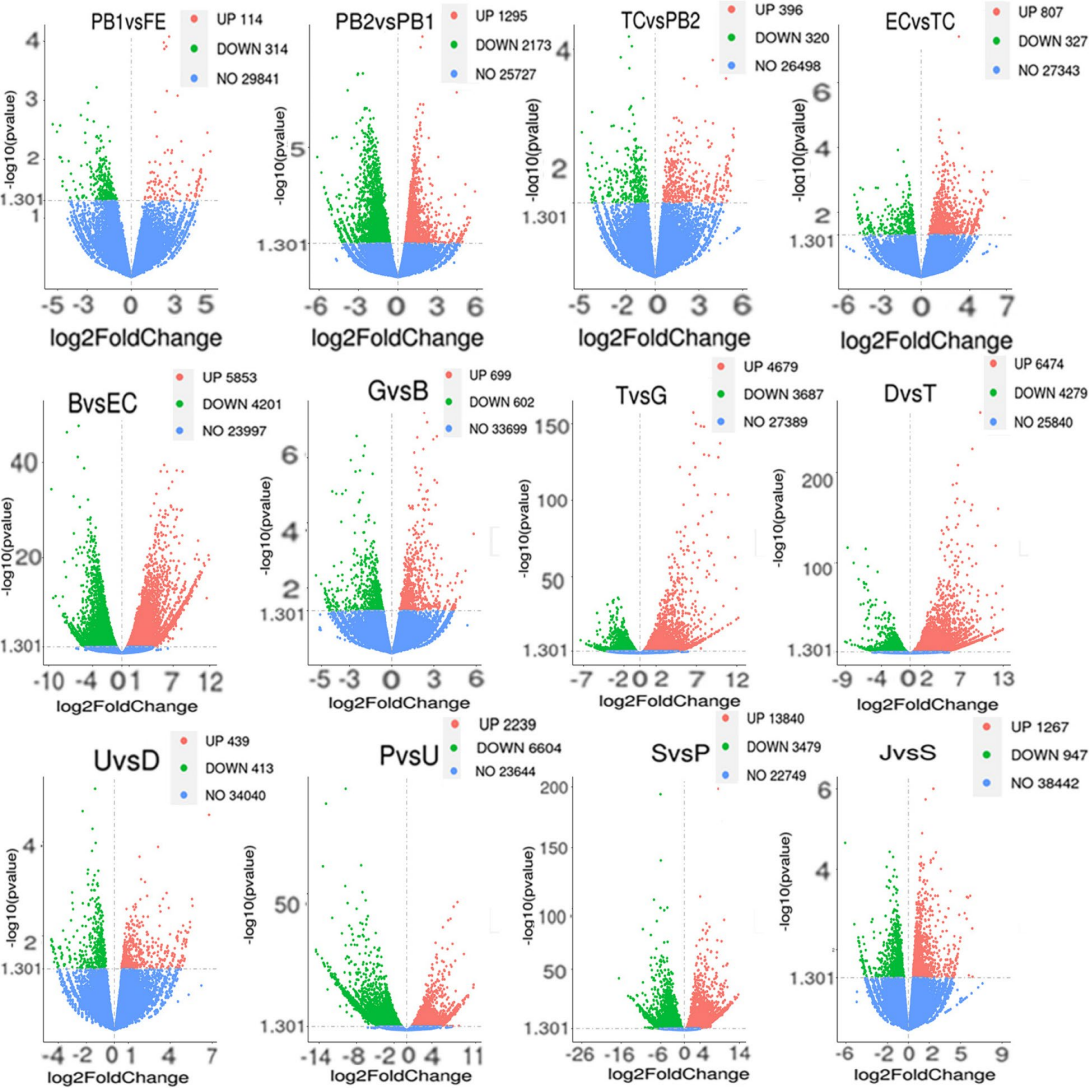
Visualization analysis of expression differences for different comparisons. DEG volcano plots of **A** FE vs. PB1; **B** PB1 vs. PB2; **C** PB2 vs. TC; **D** TC vs. EC; **E** EC vs. B; **F**: B vs. G; **G**: G vs. T; **H**: T vs. D; **I**: D vs. U; **J**: U vs. P; **K**: P vs. S; **L**: S vs. J. One dot in the volcano represents one gene, red dots represent upregulated genes, green dots represent downregulated genes, and blue dots indicate undifferentiated genes. *p*-value < 0.05, |log2FoldChange| > 0. The abscissa is log2FoldChange and the ordinate is –log10 (*p* value). The larger the value of –log10 (*p* value), the more significant the difference of the DEGs

A total of 50,284 genes were obtained after RNA sequencing, including 27,652 genes mapped to the Manila clam genome and 22,632 genes not mapped to the Manila clam genome. The mapped genes were annotated using the Pfam, GO and KEGG databases, resulting in 3,519 genes in the Pfam database, 23,196 genes in the GO database and 960 genes in the KEGG database. The heat map of all genes showed broadly consistent expression between biological duplicate samples, which implied high reliability of the sample (Fig. S4). To investigate the involvement of DEGs in the development of Manila clam, the expression levels of genes were compared between groups at different developmental stages. In total, 26,445 DEGs were obtained with |log_2_(FC)| ≥0 and *p* < 0.05. We identified significant differences in the number of DEGs for pair-wise comparisons between two adjacent periods for the 13 developmental stages. Notably, we detected more upregulated genes than downregulated genes in most samples, except PB1 vs. FE, PB2 vs. PB1, and P vs. U (Table S3). Volcano plots of the number of DEGs in the 12 comparison groups (FE vs. PB1, PB2 vs. PB1, TC vs. PB2, EC vs. TC, B vs. EC, G vs. B, T vs. G, D vs. T, U vs. D, P vs. U, S vs. P, and J vs. S) revealed a range of 114 to 13,840 significantly upregulated genes and 314 to 6604 (Fig. 2).

We performed GO and KEGG analyses to further evaluate the function of DEGs at different developmental stages. In total, 14,244 genes (28.33% of total genes) were annotated in the GO database, and 3250 genes (9.41% of total genes) were annotated in the KEGG database. The number of DEGs in the GO enrichment analysis was highly variable among the pairwise comparisons (Table 2). The number of items in the 13 samples that passed the GO terminology classification process ranged from 395 to 880. In addition, the KEGG annotation of unigenes from each group reveals that the number of pathways varies between 49 and 117compared with each group (Table 3), and a total of 4734 unigenes assigned to 181 pathways were identified (Table 3), with the number of unigenes in each pathway varying from 1 to 99.

**Table 2:** Number of DEGs in GO terms

upregulated	DEGs between different stages	DEGs upregulated	DEGs downregulated	Total number of DEGs	Biological process	Cellular components	Molecular function
1	PB1 versus FE	114	314	428	174	48	173
2	PB2 versus PB1	1295	2173	3468	403	95	262
3	TC versus PB2	396	320	716	219	44	153
4	EC versus TC	807	327	1134	338	72	199
5	B versus EC	5853	4201	10,054	472	103	295
6	G versus B	699	602	1301	344	64	208
7	T versus G	4679	3687	8366	464	103	295
8	D versus T	6474	4279	10,753	461	103	295
9	U versus D	439	413	852	294	51	182
10	P versus U	2239	6604	8843	452	96	278
11	S versus P	13,840	3479	17,319	470	106	304
12	J versus S	1267	947	2214	369	72	227

**Table 3:** Number of kegg pathway

Sample	Number of pathway	Number of significantly enriched pathways
PB1 versus FE	49	0
PB2 versus PB1	111	4
TC versus PB2	69	0
EC versus TC	76	1
B versus EC	116	7
G versus B	92	0
T versus G	117	6
D versus T	117	8
U versus D	70	2
P versus U	115	3
S versus P	117	11
J versus S	87	3

### The key DEGs involved in different developmental stages

**Fig. 3:**
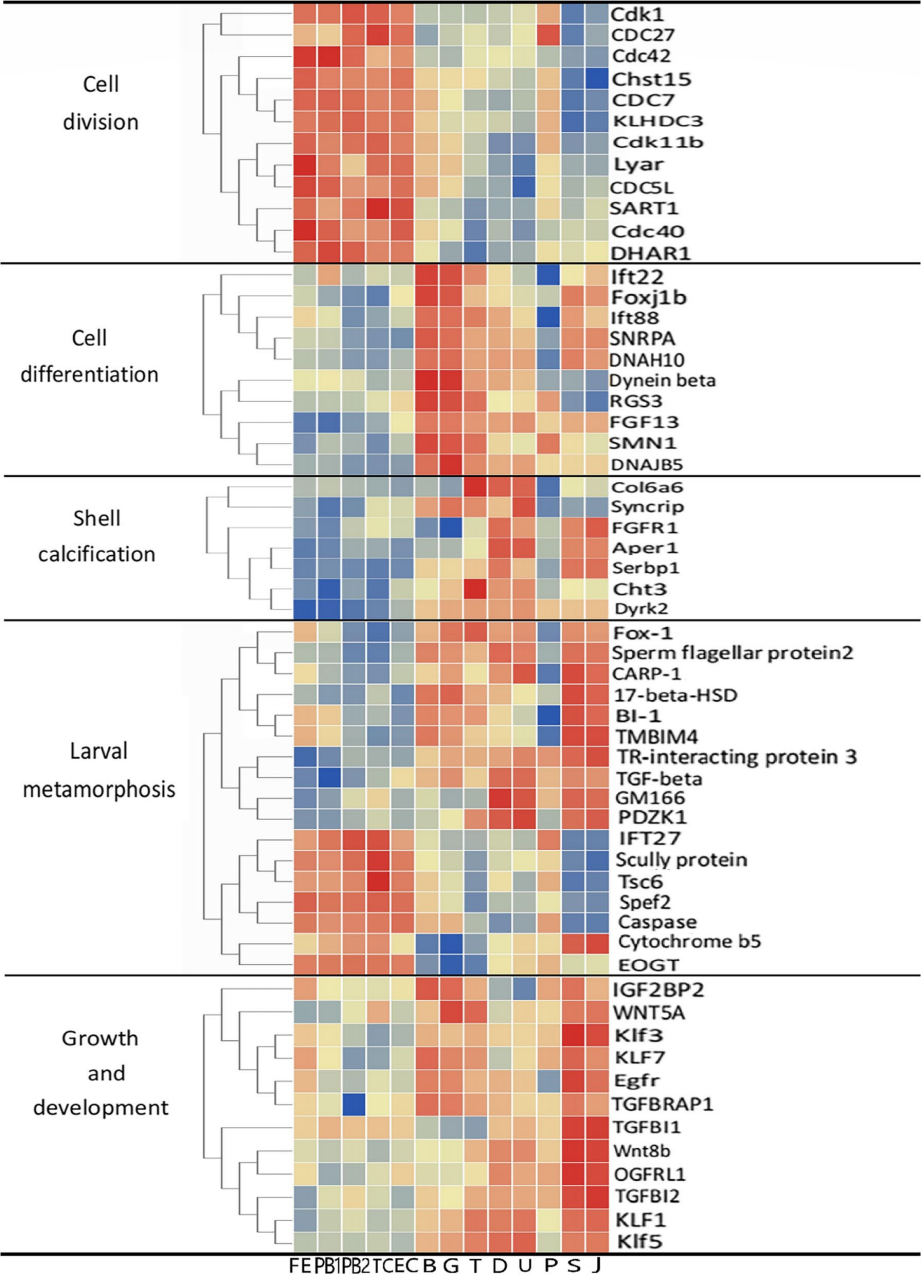
The heat map shows the expression levels of key DEGs for different molecular functions in samples from 13 different developmental periods. The color intensity from blue to red indicates the magnitude of differential expression. Red color indicates the mostly upregulated genes, and blue color indicates the mostly down regulated genes

The early development of the Manila clam was divided into different period including cell division, cell differentiation, larval growth and development, larval metamorphosis, and shell calcification (Fig. 3). Genes involved in cytokinesis were upregulated during FE-EC, including Chst15, Cdk1, Cdc40, CDC7, Lyar, Cdk11b, Cdc42, SART1, CDC27, CDC5L, DHAR1, and KLHDC3. Most genes involved in cell differentiation were upregulated during the B-G-T period, including Foxj1b, SNRPA, FGF13, SMN1, DNAJB5, Dynein beta, DNAH10, RGS3, Ift22, and Ift88. During this period, the larvae began to develop organs, and the source of nutrition changed from the yolk provided by the egg to exogenous food. During the T-D-U process, the larval shell cuticle gradually calcified until the secondary shell appeared and we found that Aper1, Cht3, Col6a6, FGFR1, Dyrk2, Syncrip, and Serbp1 were upregulated during this process. During the U-P process, the larval veliger organ was shed and the lifestyle changed from planktonic to demersal. The differential genes at work during this change were 17-beta-HSD, Cytochrome b5, Scully-protein, Sperm-flagellar-protein2, IFT27, Gm166Spef2, TR-interacting-protein-3, TGF-beta, Fox-1, EOGT, BI-1, TMBIM4, Tsc6, CARP-1, PDZK1, and Caspase. Egfr, TGFBI1, TGFBI2, wnt8b, WNT5A, IGF2BP2, OGFRL1, KLF1, Klf3, KLF7, and Klf5 were upregulated during the P-J process. Growth and development-related genes, such as EGF1 and TGFBRAP1, were consistently upregulated during the early development of Manila clam larvae.

Figure S5A indicated the important role of chitin and matrix protein-related enzyme genes during Manila clam development, and we found some genes involving formation of protein complex were up-regulated during the middle and late stages of clam larvae development. The heatmap analysis revealed gene expressions of TGF-beta gene family during S and J stages were significantly up-regulated than other periods (Fig. S5B). In addition, apoptosis-related genes and Fox transcription factors, Tyr gene changed in expression during development of Manila clam (Fig. S5C-E).

### GO analysis of DEGs

**Fig. 4:**
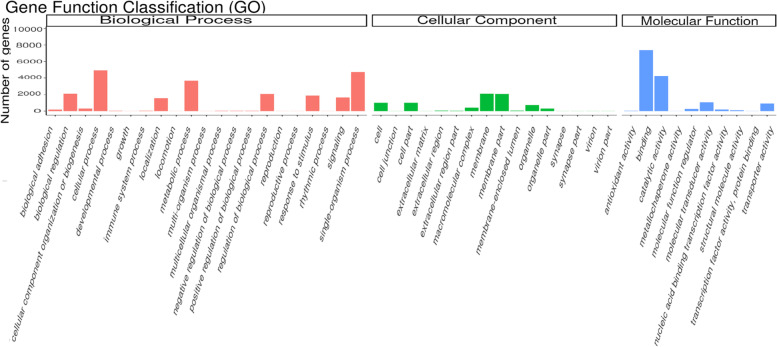
The overall GO classification annotation of the unigenes

**Fig. 5:**
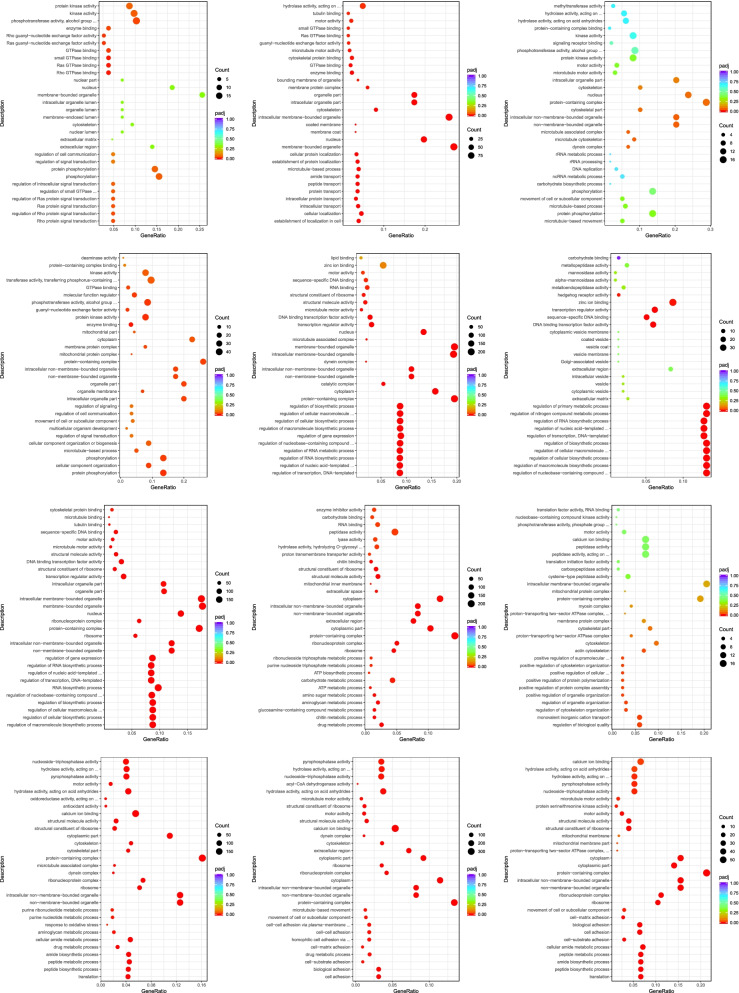
The analysis of GO functional enrichment. The ordinate represents the top 30 enriched GO terms, and the Rich factor is the proportion of the DEGs annotated in the GO term to the total annotated genes in the GO term. The deeper red the color of the Q value, the more significant the enrichment of the GO term. The significant number of DEGs in the GO term is represented by the size of the circle

Following the analysis of DEGs, we conducted GO analysis and classified the DEGs into three main categories (Fig. 4): biological processes, cellular components, and molecular functions. Next, the distribution and enrichment of DEGs in GO these functional categories were analysed to identify genomes with differential expression levels (Fig. 4). The numbers of DEGs annotated in the 12 comparison groups FE vs. PB1, PB2 vs. PB1, TC vs. PB2, EC vs. TC, B vs. EC, G vs. B, T vs. G, D vs. T, U vs. D, P vs. U, S vs. P, and J vs. S were 428, 3468, 761, 1134, 10,054, 1301, 8366, 10,753, 852, 8843, 17,319, and 2214, respectively (Fig. 5). The GO terms significantly enriched in each comparison group were Rho protein signal transduction; intracellular membrane-bound organelles; protein-containing complexes; molecular function regulator; membrane-bounded organelles; regulation of nitrogen compound metabolic process; regulation of cellular macromolecule; chitin metabolic process, chitin binding; actin cytoskeleton, regulation of biological quality; aminoglycan metabolic process, oxidoreductase activity; hydrolase activity, acyl-CoA dehydrogenase activity; and proton-transporting, motor activity, respectively. The variety of biological processes identified in the B vs. EC comparison was the largest of all comparison groups, and the cellular components and molecular functions found in S vs. P were the most diverse. The results showed that the numbers of DEGs in the FE vs. PB1 and TC vs. PB2 comparison groups were relatively low, and the numbers of DEGs in the B vs. EC and G vs. B comparison groups were significantly decreased, and the number of DEGs in the P vs. U comparison group was significantly higher than that in the other groups.

### KEGG analysis of DEGs

To understand the pathways at work at different developmental stages, we applied KEGG analysis to the DEGs (Table S4). The largest numbers of DEGs were annotated in the S vs. P comparison group, and relatively few DEGs were annotated in the FE vs. PB1 and TC vs. PB2 comparison groups. The highest numbers of pathways were found in the T vs. G, D vs. T, and S vs. P comparison groups. The lowest number of pathways was observed in the FE vs. PB1 comparison group, and the highest number of significantly enriched pathways was detected in the S vs. P comparison group.

**Fig. 6:**
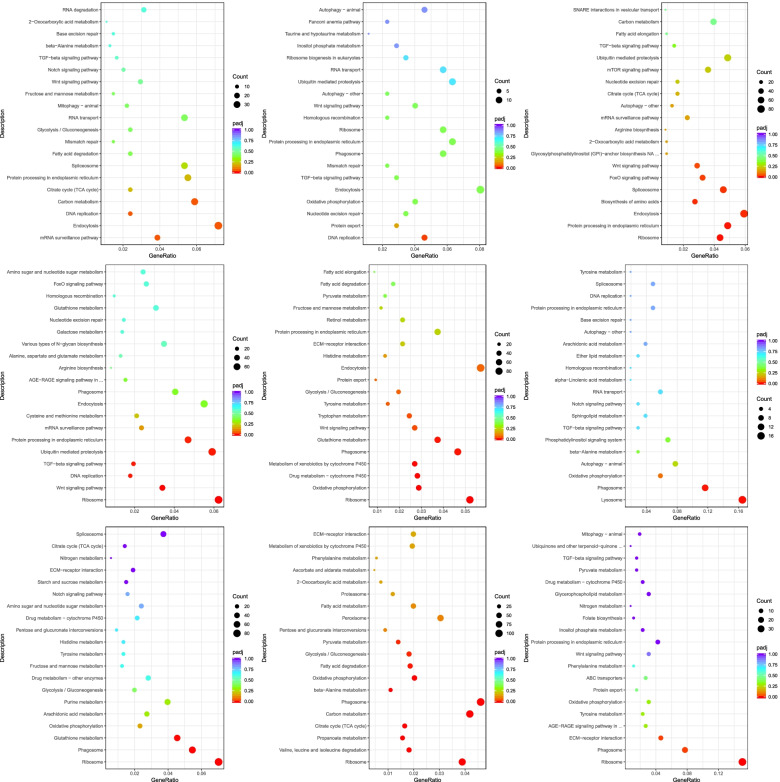
Results of KEGG pathway functional enrichment analysis. The ordinate represents the top 30 enriched KEGG pathways, and the Rich factor is the proportion of the DEGs annotated in the KEGG pathway to the total annotated genes in the KEGG pathway. The deeper red the color of the Q value, the more significant the enrichment of the KEGG pathway. The significant number of DEGs in the KEGG pathway is represented by the size of the circle.KEGG is developed by Kanehisa Laboratories and the source was cited [40–42]

The KEGG enrichment analysis results showed that a number of significantly enriched pathways played important roles in different developmental periods (Fig. 6). The KEGG enrichment analysis disclosed that eleven significantly enriched pathways (Pyruvate metabolism; Glycolysis/Gluconeogenesis; Fatty acid degradation; Oxidative phosphorylation; beta-Alanine metabolism; Phagosome; Carbon metabolism; Citrate cycle; Propanoate metabolism; Valine, leucine and isoleucine degradation; Ribosome) were observed in S vs. P comparison group, which is more than other comparison groups. The PB1 vs. PB2, EC vs. TC, B vs. EC, T vs. G, D vs. T, U vs. D, P vs. U, and J vs. S comparison groups had only four (Carbon metabolism; DNA replication; Endocytosis; mRNA surveillance pathway), one (DNA replication), six (Wnt signaling pathway; Foxo signaling pathway; Spliceosome; Biosynthesis of amino acids; Endocytosis; Protein processing in endoplasmic reticulum; Ribosome), six (Protein processing in endoplasmic reticulum; Ubiquitin mediated proteolysis; TGF-beta signaling pathway; DNA replication; Wnt signaling pathway; Ribosome ), six (Glutathione metabolism; Phagosome; Metabolism of xenobiotics by cytochrome P450; Drug metabolism - cytochrome P450; Oxidative phosphorylation; Ribosome), one(Phagosome), three (Glutathione metabolism; Phagosome; Ribosome) and two (ECM-receptor interaction; Phagosome) significantly enriched pathway, respectively.

### Weighted gene co-expression network analysis

**Fig. 7:**
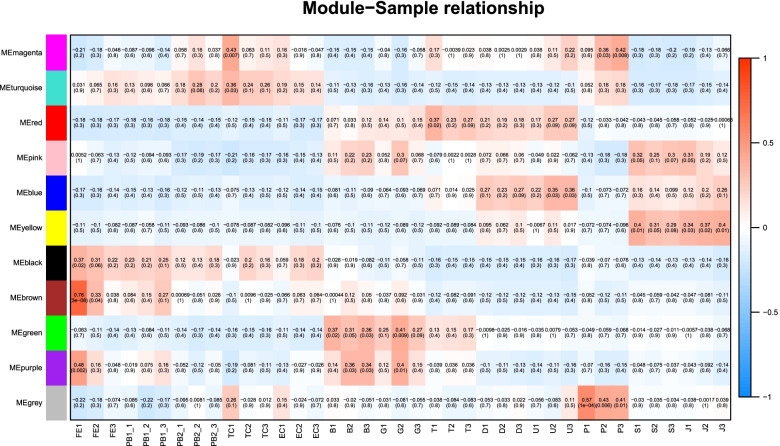
Clustering of module eigengenes and heat maps of modules in different development stages. The number in parentheses is the number of genes in each module. Blue indicates a negative correlation and red indicates a positive correlation

**Fig. 8:**
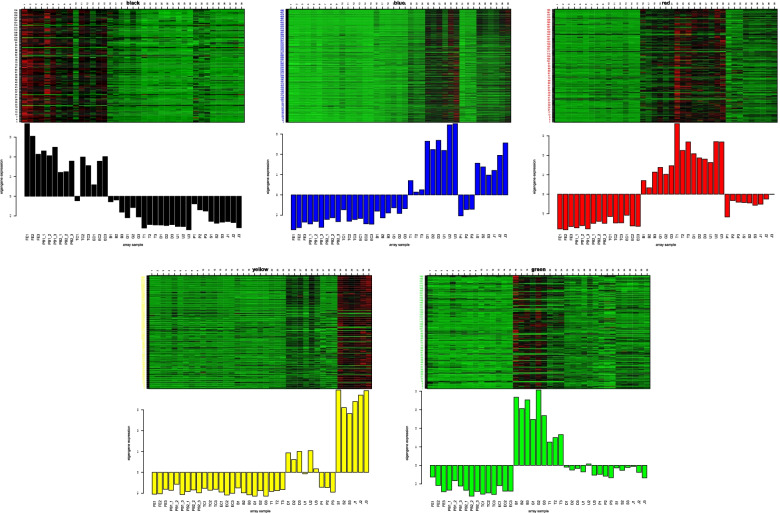
Expression of candidate genes specific to the different developmental stages in the black, blue, green, red, and yellow modules. The figure above shows a heat map of all genes assigned to the black, blue, green, red, and yellow modules at different developmental stages

We conducted weighted co-expression network analysis to investigate patterns of gene association between samples from different developmental periods. A soft threshold power of six was selected. Genes with similar expression patterns were grouped into one module among a total of 11 modules (Fig. 7). Based on the module-sample correlation analysis (Fig. S6), most of the genes in the black module are up-regulated in expression from FE to EC stage and down-regulated in expression from B to J stage. Genes in the blue module are down-regulated in expression from FE to G stage and up-regulated between T to U stage and S to J. Interestingly, in the trend of up-regulation of gene expression in the late early developmental stage, these genes are down-regulated in the P stage. In the green module, genes are down-regulated from FE1 to EC and D to J, and up-regulated from B to T. In the red module, genes are down-regulated from FE to EC with P to J and up-regulated from B to U. In the yellow module, genes are down-regulated in the FE to T and P stage and up-regulated in the D to U and S to J stage (Fig. 8).

The gene with the highest connectivity in the black module is RAD54B (DNA repair and recombination protein RAD54B, k-value = 306.3). The gene with the highest connectivity in the blue module is RPN2 (Dolichyl-diphosphooligosaccharide-protein glycosyltransferase subunit, k value = 330.02). The gene with high connectivity in the green module is Cherp (Calcium homeostasis endoplasmic reticulum protein, k value = 188.21). The gene with high connectivity in the red module is GTPBP2 (GTP-binding protein 2, k value = 318.69). The gene with high connectivity in the yellow module is ATP5A1 (ATP synthase subunit alpha, k value = 253.

### RT-qPCR confirmation of RNA-seq data

**Fig. 9:**
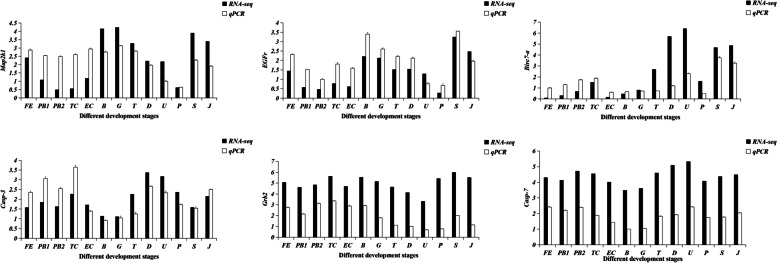
The relative mRNA expression pattern of different DEGs depending on different molecular functions at different development stages detected by qRT-PCR. The values are given in terms of relative mRNA expression. Data are presented as the means of three replicates ± standard error

To validate the sequencing data obtained from the 13 developmental period samples, we selected six genes that play an important role in the early development of Manila clams, including Mitogen-activated protein kinase (Map2k1), Epidermal growth factor receptor (Egfr), Baculoviral IAP repeat-containing protein 7-A (Birc7-a), Caspase-3 (Casp3), Growth factor receptor-bound protein 2 (Grb2), and Caspase-6 (Casp6). The trends for these genes detected by RT-qPCR were consistent with the RNA-seq data for the 13 development stages (Fig. 9), which confirmed the authenticity of our RNA-seq data.

## Discussion

### Shell calcification

During Manila clam development, calcification of the shell begins after the D- larval stage and the completion of the secondary shell during early development. A good quality secondary shell provides protection and helps the clams survive. However, shell calcification is subject to a combination of intrinsic and external factors [43]. In this study, we identified several DEGs that were involved in shell matrix formation, such as collagen [44] (a basic component of the extracellular matrix), chitin-binding proteins [45], and shell matrix proteins. Structural domain analysis showed that chitin in samples from different developmental periods contained multiple structural domains, and 37 members of the chitin family were found in Manila clam. In thecurrent study, 10 genes including chitin-related enzymes were significantly upregulated at the D vs. U and S vs. J stages (Fig. S5A). In mussels *Mytilus edulis*, chitin-related enzymes have been shown to play an important role in biochemical defense processes [46]. In a study on mollusk biomineralization, it was reported that there are many tyrosinase genes in the genome of bivalves and qPCR results showed high expression of tyrosinase genes in the mantle tissue of the Pacific oyster *C. gigas* [47]. In the present study, 15 tyrosinase genes were upregulated during Manila clam D, U, S, J and shell morphology was altered from D to U in Manila clam (Fig. S5E), indicating tyrosinase genes are likely to play important roles in the larvae shell growth in Manila clam.

### Larval metamorphosis

Larval metamorphosis is a complex biological process [48] that involves morphological and lifestyle changes [49]. During the pediveliger stage of clam larvae, the upper disc organs are shed and the lifestyle changes from planktonic to demersal; however, the detailed molecular mechanisms at work during this process are poorly understood. NO is thought to play an important role in larval metamorphosis [50], because habitat change leads to a starvation state, which inhibits NO signaling, which in turn triggers larval metamorphosis [51]. The second messenger NO plays an important role in the regulation of larval metamorphosis in marine invertebrates [52]. NO regulates larval settlement by mediating downstream calcitonin gene-related peptide signaling [53], reducing MAPK expression, and affecting extracellular signal-regulated kinase (ERK signaling), This explains the down-regulation of the MAP2K1 gene, a key gene in the MAPK pathway, during the T-U period in the results of this study (Fig. 9). Additionally, ERK phosphorylation levels are required for transcription of key degenerate genes [54]. In bivalves, such as *C. gigas* and *M. coruscus*, NO has been shown to be a negative regulator of larval metamorphosis [55]. An important role for γ-aminobutyric acid in larval metamorphosis has also been reported [56], and γ-aminobutyric acid functions internally as a neurotransmitter to control the initiation of metamorphosis.

### Transforming growth factor-beta (TGF-β)

TGFs constitute a superfamily of growth factors with important functions in cell growth, proliferation, differentiation, and apoptosis [57]. Activation of the TGF-β receptor is followed by intracellular effector-Smad protein translocation into the nucleus [58, 59]. In our study, three TGF-βs were significantly upregulated during the B and G stages of Manila clam development, which indicated an important role for these genes during the period of cell differentiation [60]. TGF family analysis revealed that Manila clams contained nine TGF superfamily members, and these genes were expressed in different patterns in the 13 developmental stages (Fig. S5B). An important role of TGF-β in the development of the Pacific oyster gonad has been reported, as its specific expression in gonads peaked when the gonads reached full maturity [61]. Two TGF-β proteins in *Drosophila* act as heterodimers to stimulate male germ cell proliferation [62]. In Tilapia (*Oreochromis mossambicus*), TGF-β ligands exhibit tissue specificity in the gonads, and the expression profiles of eight genes in the gonads at different stages of development suggest an important role for TGF-β ligands in sex determination and reproduction [63]. However, the role of TGF-β in the growth of Manila clams requires further study.

### Apoptosis

The apoptotic program is a highly conserved process that is widespread in nature [64], and it is thought to be closely related to biological evolution. During sea urchin embryonic development, apoptotic cells are found in the flagellar zone prior to larval metamorphosis [65]. It has been reported that apoptosis is induced in larval epithelial cells during accelerated larval metamorphosis [66]. Caspase is closely associated with apoptosis, and caspases were shown to play a key role in foot loss during larval metamorphosis of Fujian oyster (*C. angulata*) [67]. In this study, apoptosis-inducing factors and apoptosis protein-related genes were consistently expressed at almost every period (Fig. 9, Fig. S5D), suggesting that apoptosis factors were involved in the early development of Manila clams. Apoptosis is also an important part of the host defense system [68]. However, in the paralytic shellfish toxin induced hemocytes death process, the caspase-dependent pathway was found to induce apoptosis in Pacific oyster immune cells, and the resistance of the oysters to microbial infection diminished [69]. In our study, we identified 47 members of the Manila clam caspase structural domain using hmm search. A study in which pre-metamorphic *M. meretrix* larvae were treated with Caspase inhibitors showed that apoptosis may be the main mechanism of veliger degeneration during metamorphosis, and in situ labelling showed that caspases were involved in the morphological change process of the larvae [70]. Thus, the role of caspase in the metamorphosis of Manila clam larvae is worthy of further study.

### FOXO transcription factor

We found that the FOXO pathway was significantly expressed during the EC to B transition, when the number of upregulated DEGs was 37 and the number of downregulated DEGs was nine. The results indicated that FOXO transcription factors were involved in the EC-B process, during which cell proliferation mainly occurred. FOXO proteins were previously reported to be involved in cellular processes, including cell proliferation and apoptosis [71]. FOXO transcription factors also function as transcriptional activators, and their activity is inhibited by insulin and growth factor signaling [72]. This behaviour explains the observed upregulation of FOXO transcription factor expression during the blastula stage and the gastrula stage. FOXO factors play a role in regulating cell clearance mechanisms, and FOXO3 induces the expression of several autophagy genes involved in the coordination of autophagy-related gene networks [73]. FOXO factors also regulate the ubiquitin-proteasome system [74]. Thus, FOXO is likely to play important roles in the maintenance of cellular homeostasis during the early cell division period in Manila clams.

### Glutathione metabolism pathway

Glutathione is a common antioxidant enzyme in bivalves and is used as an important metabolite for protection against oxidative stress in cells [75]. In this study, the glutathione metabolic pathway was significantly enriched during the P. vs. U period versus the T. vs. G. G to T period. Manila clam larvae undergo organogenesis. Some studies have revealed the role of glutathione as more than an antioxidant; the ability of cysteine residues of GSH to be reversibly oxidized in various protein targets acts as a mediator of many processes [76] such as cell proliferation, cell differentiation, and cell death [77, 78]. These studies explain the significant enrichment of the glutathione metabolic pathway during the T to G period, when manila calm undergoes cell differentiation and forms larvae. Glutathione has been found to promote growth in aquatic animals, such as pearl oyster (*P. fucata martensii*) [79], and white shrimp (*Litopenaeus vannamei*) [80].

### DNA repair and recombination protein RAD54B

Weighted gene co-expression network analysis showed that RAD54B was the gene with the highest k-value in the black module and, in general, genes with the highest connectivity (k-value) ranking can be considered hub genes [81]. Rad54B is a DNA-dependent ATPase that has been described as being involved in recombinant repair of DNA damage [82]. Many RAD homologs for longevity, neurological and immune adaptation have been identified in the lobster genome, including seven homologs of RAD54 [83]. This implies a potential role for RAD genes in biological stress resistance. In the present study, the RAD54B gene was up regulated from FE to EC, indicating that the RAD54B gene has an important role in fertilized egg development in the Manila clam, but the exact function needs further study.

## Conclusion

In this study, we analyzed 13 stages of early development in the Manila clam by RNA-seq, including Fertilized egg, 1 st polar body, 2 st polar body, Two-cell, Eight-cell, Blastula, Gastrula, Trochophora, D-larva, Umbo-veliger, Pediveliger, Single pipe juvenile, and Juvenile. We found that a unique gene expression pattern in each stage, and similar expression profile was observed in adjacent stages. The development process of Manila clam is broadly divided into cell division, cell differentiation, larval growth and development, larval metamorphosis, and shell calcification. The rho protein signaling, membrane-bound organelle, were prominent at the early development of Manila clam. KEGG analysis showed that the stage of Manila clam cell differentiation (EC-B) was associated with the Foxo pathway and Manila clam hatching (G-T) was associated with the TGF-beta signaling pathway. In addition, the stage of shell alteration (T-D) is associated with Tyrosine metabolism. These data provide insights into the molecular mechanisms of early Manila clam development and may help to enhance Manila clam reproduction and artificial breeding.

## Supplementary Information

**Additional file 1.**

**Additional file 2.**

**Additional file 3.**

**Additional file 4.**

**Additional file 5.**

**Additional file 6.**

**Additional file 7.**

**Additional file 8.**

**Additional file 9.**

**Additional file 10.**

## Data Availability

All data generated or analyzed during this study are included in this published article. The raw sequences for R. philippinarum have been deposited in the NCBI PRJNA808620 (https://www.ncbi.nlm.nih.gov/bioproject/PRJNA808620).
