# Supplementary figures and images for: Comparative transcriptomic analysis revealed dynamic changes of distinct classes of genes during development of the Manila clam (Ruditapes philippinarum)
Source: BMC Genomics. 2022 Sep 29;23:676. doi: 10.1186/s12864-022-08813-0 (PMC9524096; doi:10.1186/s12864-022-08813-0)

FE

G

U

J


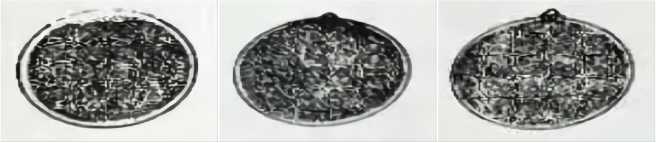

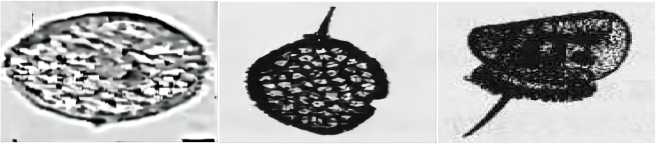

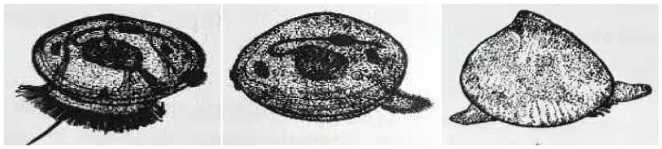

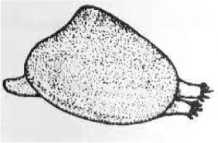


PB1

PB2


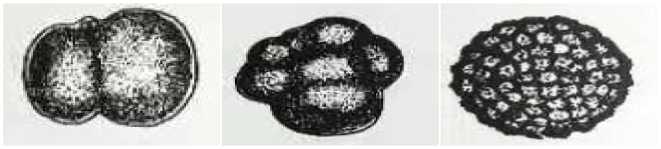


TC

EC

B

T

D

S

P

Figure S1. Morphology of the Manila clam during 13 stages of development.

Supplement: Supplementary file 1 — Additional file 1. [file 12864_2022_8813_MOESM1_ESM.docx]

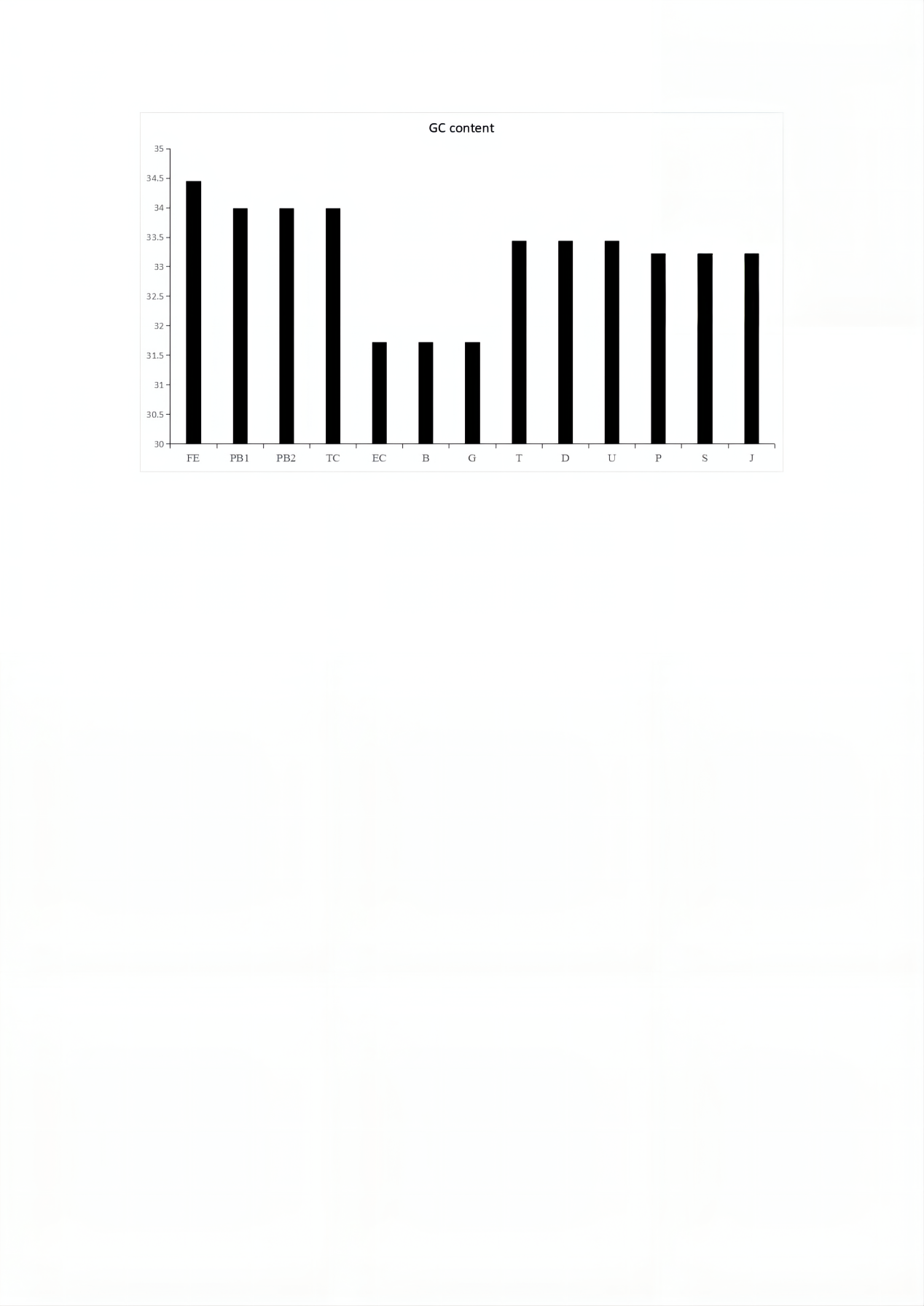


Figure S2. GC content levels of 13 development stages.

Supplement: Supplementary file 2 — Additional file 2. [file 12864_2022_8813_MOESM2_ESM.docx]

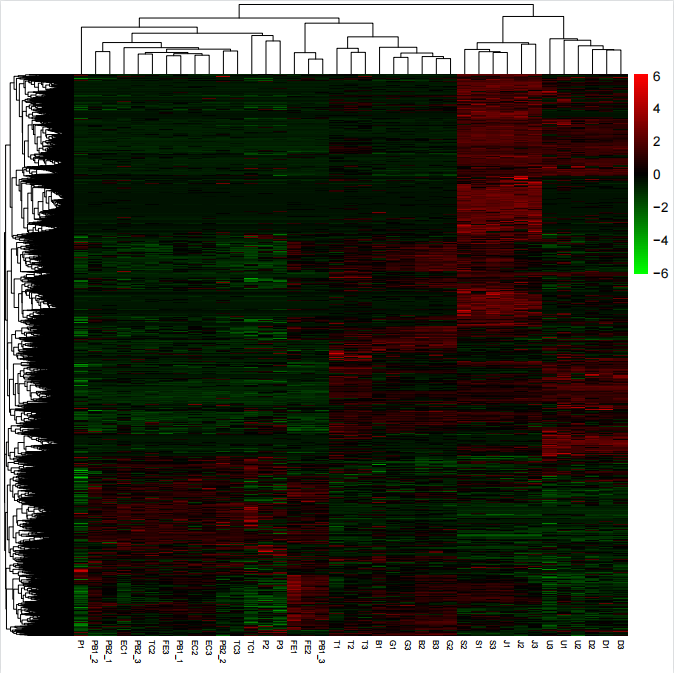
Figure S4. Heatmaps for all genes expression level of 13 development stages.

Supplement: Supplementary file 4 — Additional file 4. [file 12864_2022_8813_MOESM4_ESM.docx]
