# Supplementary material for: Comparative transcriptomic analysis revealed dynamic changes of distinct classes of genes during development of the Manila clam (Ruditapes philippinarum)
Source: BMC Genomics. 2022 Sep 29;23:676. doi: 10.1186/s12864-022-08813-0 (PMC9524096; doi:10.1186/s12864-022-08813-0)
Supplement: Supplementary file 3 — Additional file 3. [file 12864_2022_8813_MOESM3_ESM.docx]

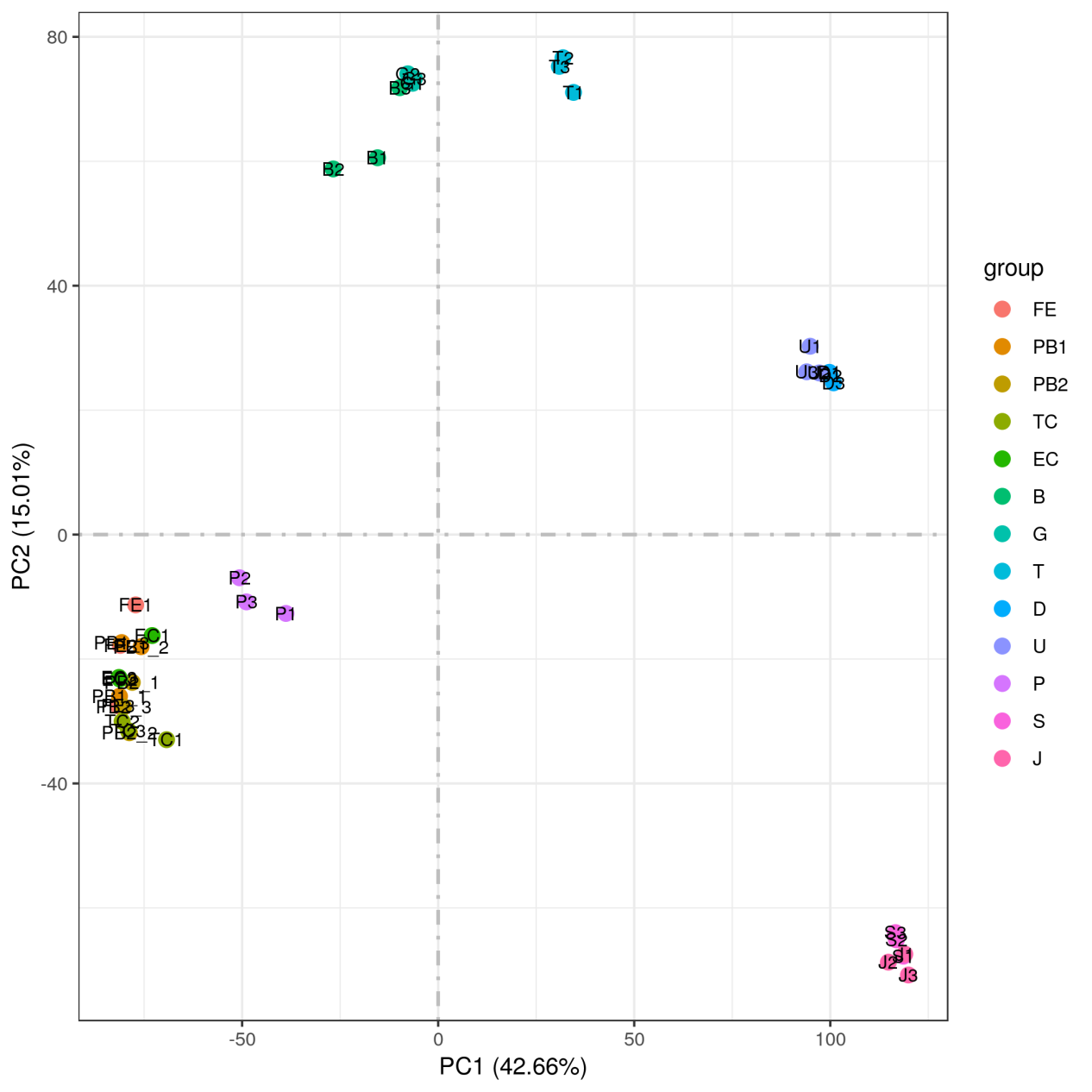
Figure S3. Principal component analysis (PCA) plot of transcriptional expression of 13 development stages.
