# Supplementary material for: Comparative transcriptomic analysis revealed dynamic changes of distinct classes of genes during development of the Manila clam (Ruditapes philippinarum)
Source: BMC Genomics. 2022 Sep 29;23:676. doi: 10.1186/s12864-022-08813-0 (PMC9524096; doi:10.1186/s12864-022-08813-0)
Supplement: Supplementary file 5 — Additional file 5. [file 12864_2022_8813_MOESM5_ESM.docx]

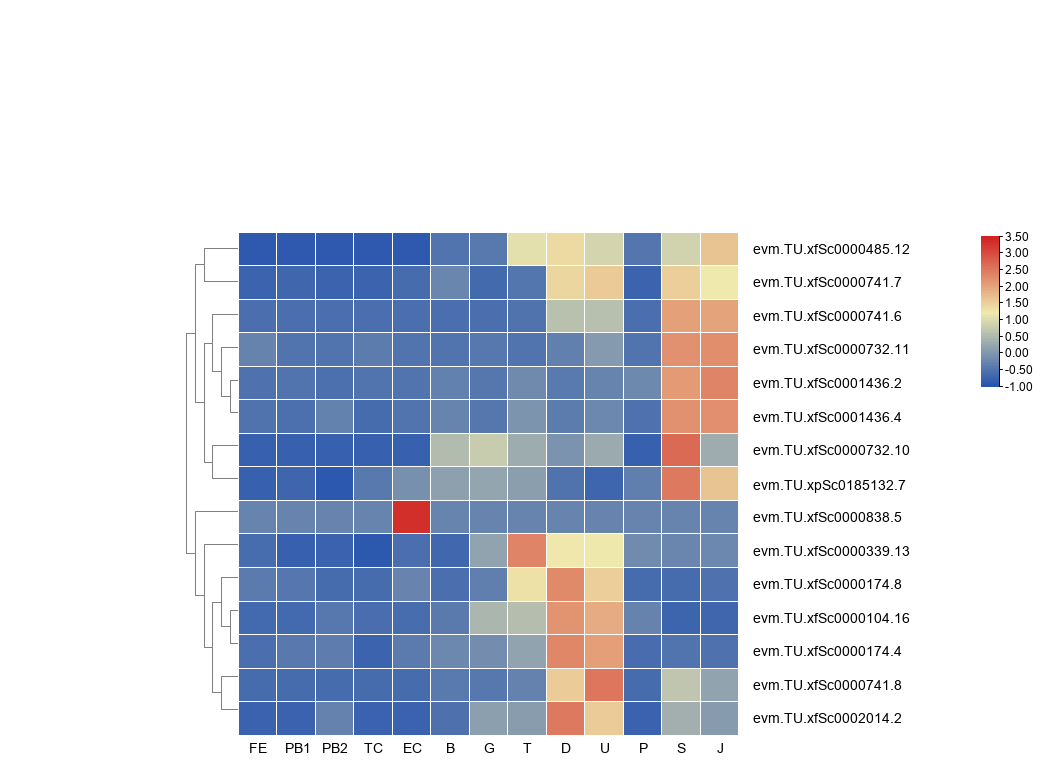

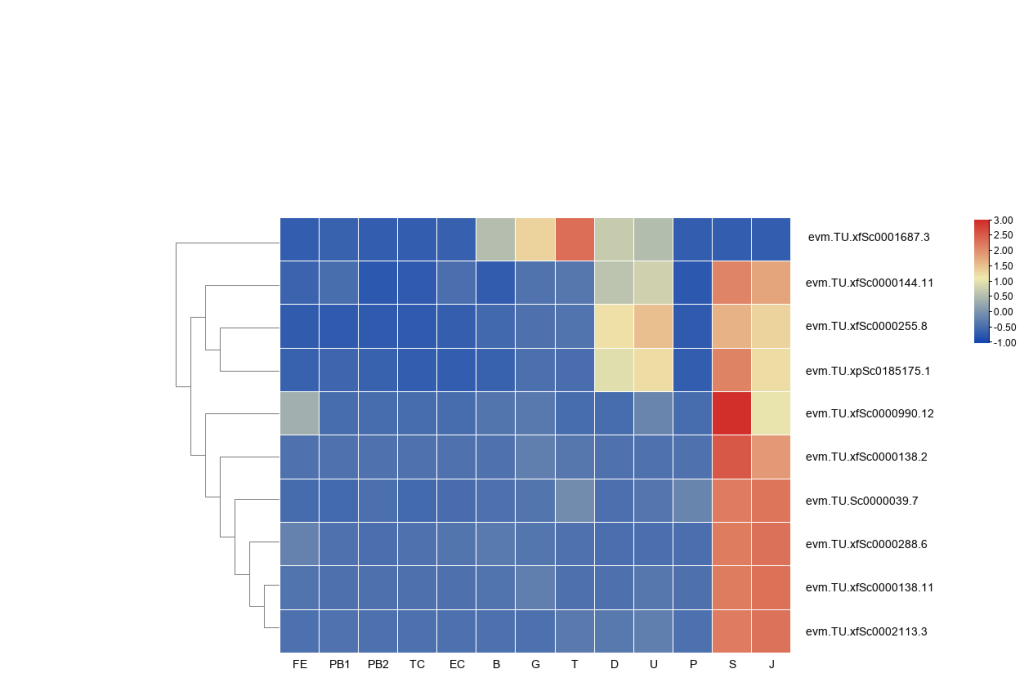

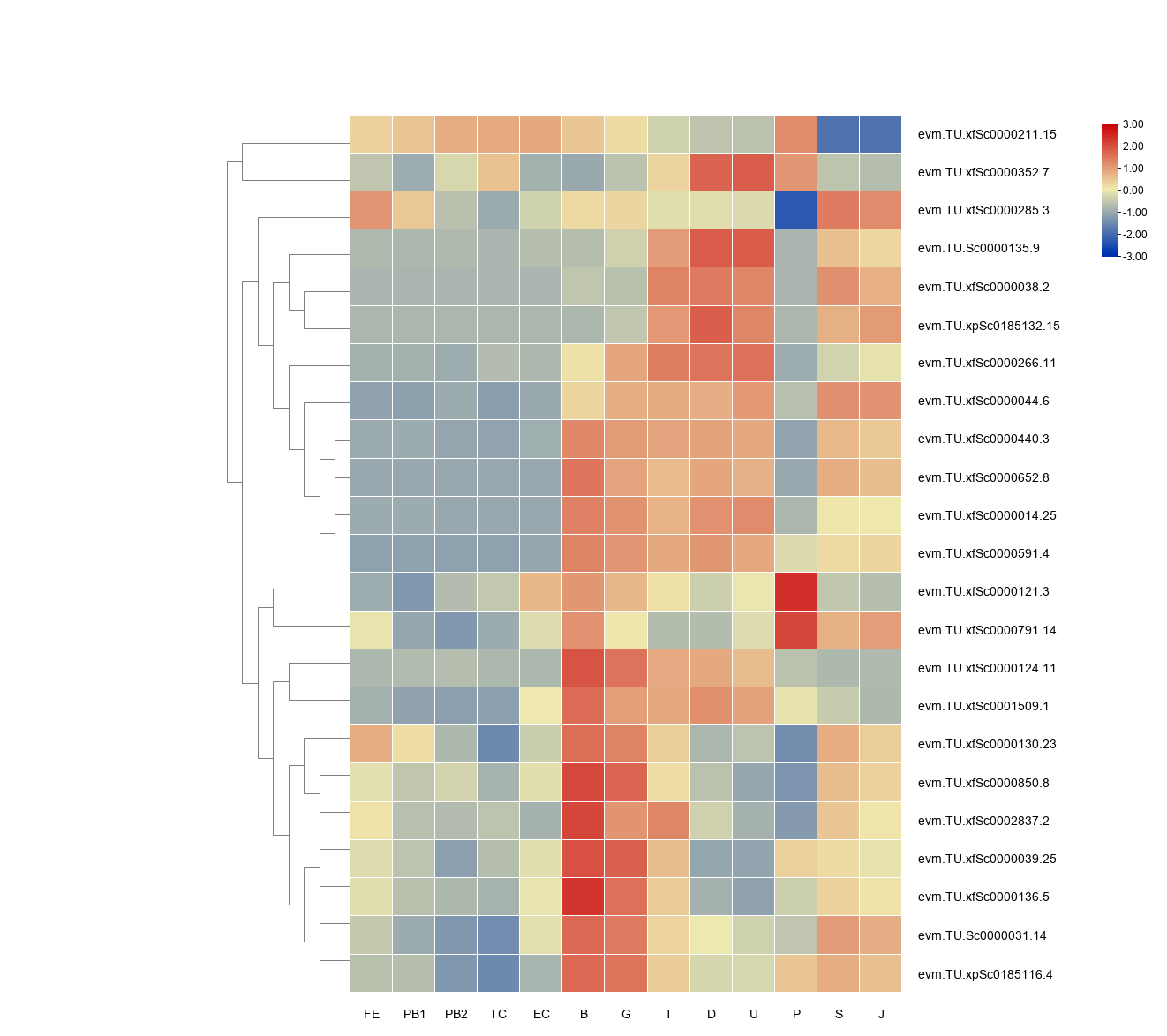

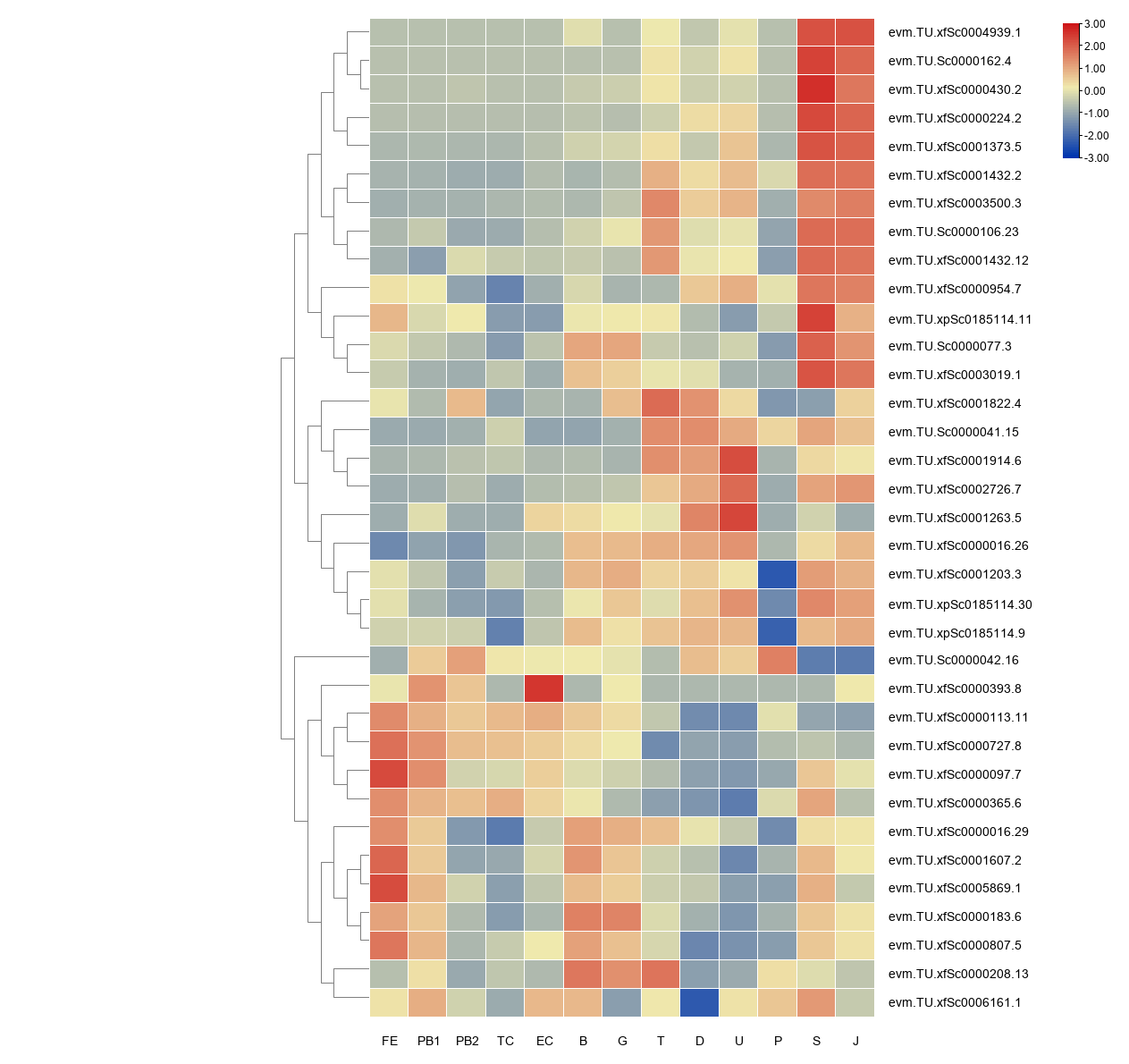

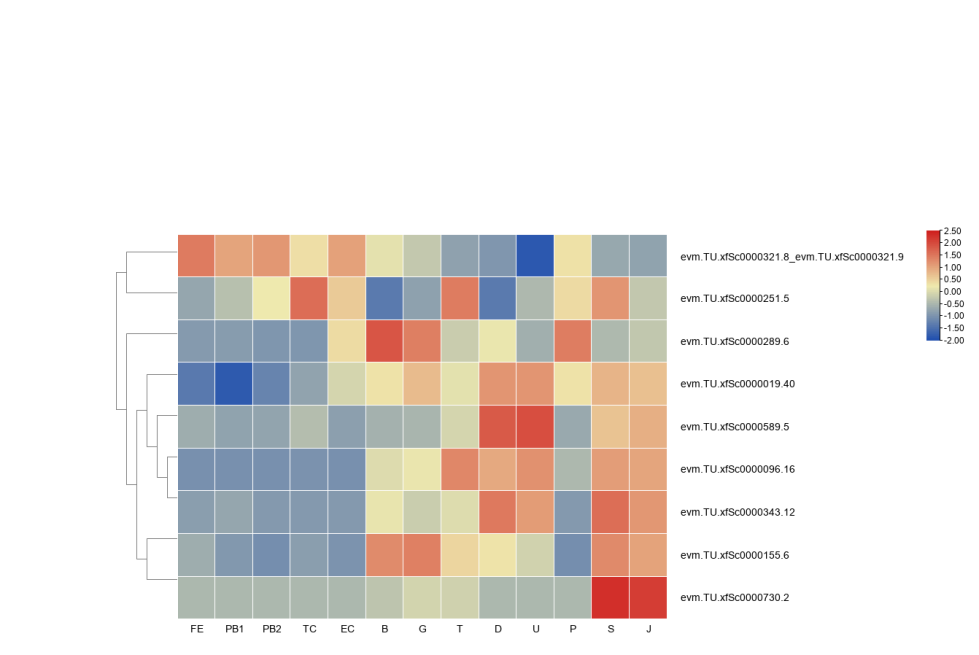


**ER**

**D**

**C**

**B**

**A**

Fig. S5A: Heatmaps for chitin gene expression level of 13 development stages.

A

Fig. S5B: Heatmaps for TGF-beta family expression level of 13 development stages.

Fig. S5C: Heatmaps for Fox gene expression level of 13 development stages.

Fig. S5D: Heatmaps for Apoptosis gene expression level of 13 development stages.

Fig. S5E: Heatmaps for Tyr gene expression level of 13 development stages.
