# Supplementary material for: Comparative transcriptomic analysis revealed dynamic changes of distinct classes of genes during development of the Manila clam (Ruditapes philippinarum)
Source: BMC Genomics. 2022 Sep 29;23:676. doi: 10.1186/s12864-022-08813-0 (PMC9524096; doi:10.1186/s12864-022-08813-0)
Supplement: Supplementary file 7 — Additional file 7. [file 12864_2022_8813_MOESM7_ESM.docx]

| Sample order | Abbreviation | Full name | Sampling time |
| --- | --- | --- | --- |
| 1 | FE | Fertilized egg | 0min |
| 2 | PB1 | 1 st polar body | 15min |
| 3 | PB2 | 2 st polar body | 20min |
| 4 | TC | Two-cell | 1h |
| 5 | EC | Eight-cell | 1h 30min |
| 6 | B | Blastula | 3h |
| 7 | G | Gastrula | 6h |
| 8 | T | Trochophora | 12h 30min |
| 9 | D | D-larva | 19h |
| 10 | U | Umbo-veliger | 2d |
| 11 | P | Pediveliger | 15d |
| 12 | S | Single pipe juvenile | 19d |
| 13 | J | Juvenile | 21d |

Table S1 Full names and sampling order of 13 samples on Manila clam
