# Supplementary material for: Comparative transcriptomic analysis revealed dynamic changes of distinct classes of genes during development of the Manila clam (Ruditapes philippinarum)
Source: BMC Genomics. 2022 Sep 29;23:676. doi: 10.1186/s12864-022-08813-0 (PMC9524096; doi:10.1186/s12864-022-08813-0)
Supplement: Supplementary file 8 — Additional file 8. [file 12864_2022_8813_MOESM8_ESM.docx]

| Gene ID | Gene | Gene name | Oligonucleotide (5′-3′) |
| --- | --- | --- | --- |
| evm.TU.xfSc0003039.1 | Birc7-a | Baculoviral IAP repeat-containing protein 7-A | F: ATCCGCCGACTTTTCAATTTGTTCTG |
|  |  |  | R: GCCAGACGAATGAAAGGAGATGAATTG |
| evm.TU.xfSc0000102.17 | Casp7 | Caspase-7 | F: AGGGAGGGCGATAATCATAAACAACAG |
|  |  |  | R: ACCTCGAAACCCATGTCCATAAATCTC |
| evm.TU.xfSc0000182.13 | CASP3 | Caspase-3 | F: ACAATAGTGGACATAGCAGCGTGTG |
|  |  |  | R: TGCTGGTCAAGTAGCTAAGCGAAC |
| evm.TU.xfSc0001343.1 | Egfr | Epidermal growth factor receptor | F: AACTCTGGAGAATGACACTGTTCGATG |
|  |  |  | R: CCAAGCATAGATGGTTACATGGTTGAC |
| evm.TU.xfSc0000570.18 | Grb2 | Growth factor receptor-bound protein 2 | F: AGGCGAAATGAAGCCGAGGAATTG |
|  |  |  | R: CTCCTGGCGCACTTTCACTGTG |
| evm.TU.xfSc0000201.7 | MAP2K1 | Mitogen-Activated Protein Kinase Kinase | F: GCTTCAGCCTTCTTCACAAATGGATG |
|  |  |  | R: ATTGTTAACGAGCCGCCACCTAC |

Table S2 Combination of primers used in qRT-PCR assays.
