# Supplementary material for: Comparative transcriptomic analysis revealed dynamic changes of distinct classes of genes during development of the Manila clam (Ruditapes philippinarum)
Source: BMC Genomics. 2022 Sep 29;23:676. doi: 10.1186/s12864-022-08813-0 (PMC9524096; doi:10.1186/s12864-022-08813-0)
Supplement: Supplementary file 9 — Additional file 9. [file 12864_2022_8813_MOESM9_ESM.docx]

Table.S3 Number of up and down DEGs with 13 development stages.

| Sample | DEGs Number | Up | Down |
| --- | --- | --- | --- |
| FE vs PB1 | 428 | 114 | 314 |
| PB2 vs PB1 | 3468 | 1295 | 2173 |
| TC vs PB2 | 716 | 396 | 320 |
| EC vs TC | 1134 | 807 | 327 |
| B vs EC | 10054 | 5853 | 4201 |
| G vs B | 1301 | 699 | 602 |
| T vs G | 8366 | 4679 | 3687 |
| D vs T | 10753 | 6474 | 4279 |
| U vs D | 852 | 439 | 413 |
| P vs U | 8843 | 2239 | 6604 |
| S vs P | 17319 | 13840 | 3479 |
| J vs S | 2214 | 1267 | 947 |
