# Supplementary material for: Comparative transcriptomic analysis revealed dynamic changes of distinct classes of genes during development of the Manila clam (Ruditapes philippinarum)
Source: BMC Genomics. 2022 Sep 29;23:676. doi: 10.1186/s12864-022-08813-0 (PMC9524096; doi:10.1186/s12864-022-08813-0)
Supplement: Supplementary file 10 — Additional file 10. [file 12864_2022_8813_MOESM10_ESM.docx]

Table S4 Number of DEGs in kegg pathway analysis.

| Sample | KEGG Number | DEGs Number | Number of significantly enriched pathways |
| --- | --- | --- | --- |
| FE vs PB1 | 49 | 89 | 0 |
| PB2 vs PB1 | 111 | 910 | 4 |
| TC vs PB2 | 69 | 112 | 0 |
| EC vs TC | 76 | 262 | 1 |
| B vs EC | 116 | 2253 | 7 |
| G vs B | 92 | 317 | 0 |
| T vs G | 117 | 1953 | 6 |
| D vs T | 117 | 2669 | 8 |
| U vs D | 70 | 185 | 2 |
| P vs U | 115 | 1971 | 3 |
| S vs P | 117 | 3915 | 11 |
| J vs S | 87 | 375 | 3 |
